# Highly Efficient One-Step Conversion of Fructose to Biofuel 5-Ethoxymethylfurfural Using a UIO-66-SO_3_H Catalyst

**DOI:** 10.3389/fchem.2022.900482

**Published:** 2022-05-09

**Authors:** Kangyu Zhao, Yanping Xiang, Xiaoao Sun, Linjiao Chen, Jiafu Xiao, Xianxiang Liu

**Affiliations:** ^1^ National and Local Joint Engineering Laboratory for New Petro-chemical Materials and Fine Utilization of Resources, Key Laboratory of the Assembly and Application of Organic Functional Molecules of Hunan Province, Hunan Normal University, Changsha, China; ^2^ Hunan Provincial Key Laboratory for Synthetic Biology of Traditional Chinese Medicine, School of Pharmaceutical Sciences, Hunan University of Medicine, Huaihua, China

**Keywords:** biomass, fructose, 5-ethoxymethylfurfural, catalysis, one-step conversion

## Abstract

In this study, a novel sulfonic acid-modified catalyst for MOFs (UIO-66-SO_3_H) was synthesized using chlorosulfonic acid as a sulfonating reagent and first used as efficient heterogeneous catalysts for the one-pot conversion of fructose into biofuel 5-ethoxymethylfurfural (EMF) in a cosolvent free system. The physicochemical properties of this catalyst were characterized by Fourier transform infrared spectroscopy (FT-IR), transmission electron microscopy (TEM), and powder X-ray diffraction (XRD). The characterization demonstrated that the sulfonic acid group was successfully grafted onto the MOF material and did not cause significant changes to its morphology and structure. Furthermore, the effects of catalyst acid amount, reaction temperature, reaction time, and catalyst dosage on reaction results were investigated. The results showed that the conversion of fructose was 99.7% within 1 h at 140°C, while the EMF yield reached 80.4%. This work provides a viable strategy by application of sulfonic acid-based MOFs for the efficient synthesis of potential liquid fuel EMF from renewable biomass.

## Introduction

As the only renewable carbon resource with extensive distribution and abundant reserves in nature, biomass has become the most attractive potential energy to replace fossil fuels (Hu et al., 2015; Tang et al., 2019; Guo D. et al., 2020), especially in recent years, with the shortage of fossil resources and the environmental problems brought by the applications, the development of new methods for the conversion of biomass and its platform molecules into fuels and chemicals is a current priority (Liu et al., 2016; Venkata Mohan et al., 2016).

The products gained from biomass conversion mainly include polyols, furans, organic acids and their ester derivatives, short-chain alkanes, and other basic platform chemicals and new fuels. The large-scale utilization of these biomass conversion products will play a significant leading role in sustainable and healthy socio-economic development (Hommes et al., 2019; He 2021; Hoang and Pham, 2021). The acid-catalyzed conversion of biomass to biomass platform molecule 5-hydroxymethylfurfural (HMF) has been previously reported in some research and progress (Zhao et al., 2007; Guo H. et al., 2020; Liu et al., 2021; Yin et al., 2021), and its derivative 5-ethoxymethylfurfural (EMF) is also a potential fuel or fuel additive with excellent properties such as high energy density and low toxicity, with energy density up to 8.7 kWh/L, even higher than ethanol (6.1 kWh/L), and has good oxidation properties when used as a fuel (Chen et al., 2019a; Guo D. et al., 2020). Currently, the main synthesis pathway of EMF is based on biomass sugar compounds (e.g., glucose, fructose, and cellulose), which are converted to EMF in an ethanol system through acid catalysis (Chen et al., 2019b; Dai et al., 2019; Zhang et al., 2020). Among them, fructose exists in large quantities in the free state in the pulp of fruits and honey and is a common biomass raw material. As a reaction substrate for the generation of EMF, unlike glucose and cellulose, fructose has a strong crystal structure, is easily soluble in conventional organic solvents, and has a higher yield than glucose and cellulose (Zhang et al., 2019). Meanwhile, the acidic catalyst has a crucial role in the synthesis of the EMF reaction, making the conversion process more complete and efficient. The catalysts reported in previous studies are zeolite catalysts, sulfonic acid-type solid acid catalysts, and heteropolyacids (Li et al., 2016; Liu et al., 2016; Kumari et al., 2019; Xiang et al., 2021). In particular, sulfonic acid-type catalysts have high protonic acid strength, which is beneficial for the synthesis of EMF. For instance, Zhang et al (2018) reported sulfonated organic hyper-cross-linked polymer (HCP)-based carbohydrate catalysts to yield 78.9% EMF, 15.4% HMF, and 4.6% ethyl levulinate (EL) in an ethanol-dimethyl sulfoxide system with fructose as feedstock. Yan et al (2022) found sulfonic acid-based annealed functionally biobased carbon microspheres loading polytetrafluoroethylene (A-BCMSs-SO_3_H@PTFE) catalyst applied to the acid-catalyzed synthesis of liquid EMF. The yield of EMF could reach more than 36.6% after 72 h of the acid-catalyzed reaction. Manjunathan et al (2021) used the sulfonated hydrophobic mesoporous organic polymer (MOP-SO_3_H) in the production of EMF by the one-pot method using fructose in an ethanol solvent. The conversion of fructose was 99.3%, and the EMF yield reached 72.2% at 100°C for 5 h. These results show that loading acid-functionalized sulfonic acid groups onto the carrier can produce sulfonic acid-based solid acid catalysts to improve the efficiency of fructose conversion to EMF (Nakajima and Hara 2012). Therefore, finding a suitable, modified as well as stable performance carrier is a prerequisite for successful catalyst preparation.

**FIGURE 1:**
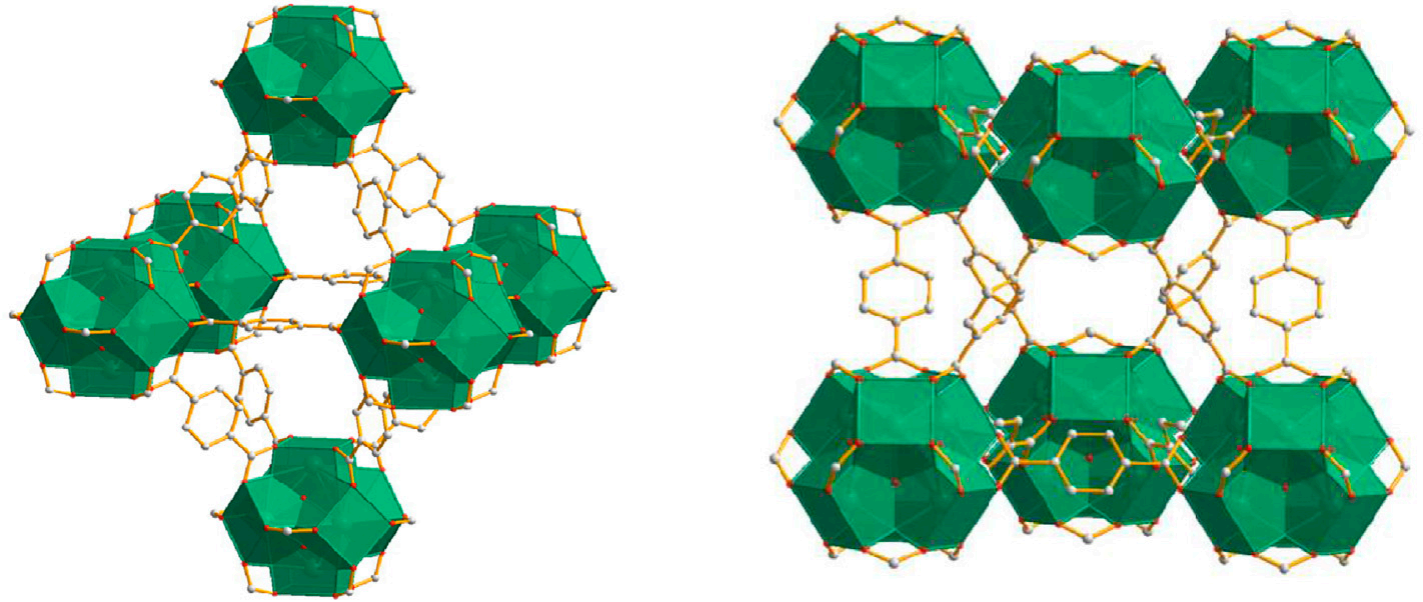
Structure of UIO-66 (Guillerm et al., 2012; Olorunyomi et al., 2021). Gray and red balls represent carbon and oxygen atoms, respectively. Zirconium octahedral is in green. Hydrogen atoms are omitted for clarity.

Metal–organic framework (MOF) materials, developed rapidly in the last two decades, have a large ordered specific surface area, adjustable pore size, customizable functions, and a large number of active junctions (Herbst and Janiak 2017; Lv et al., 2019). As a new type of MOFs, UIO-66 has great stability and can exist stably in an acidic environment, and UIO-66 is a hydrophobic material with the molecular formula [Zr_6_(OH)_4_O_4_(BDC)_6_], which is a Zr_6_-cluster as the central structural unit and 12 terephthalic acid (H_2_BDC) as the ligand to form a three-dimensional topology of metal–organic backbone materials with the structure shown in Figure 1 (Guillerm et al., 2012; Olorunyomi et al., 2021). Also, UIO-66 is a hydrophobic material which is well suited for the conversion of biobased feedstock (Lv et al., 2019; Oozeerally et al., 2021). It also inherits the characteristics of ordinary MOF materials such as large specific surface area, adjustable pore size, and easy modification, which is a good catalyst supporter.

Based on the aforementioned details, in this study, we report a sulfonic acid-functionalized UIO-66-type MOFs (UIO-66-SO_3_H) prepared under mild conditions. The catalytic performance of these catalysts was investigated for selective preparation of EMF from fructose in the one-pot strategy. Also, the effects of reaction temperature, reaction time, catalyst dosage, and acid amount on the yield of EMF were also evaluated. In addition, the morphology and physicochemical properties of the catalysts were characterized and discussed.

## Experimental

### Materials

Terephthalic acid was purchased from Aladdin Industrial Corporation. 5-Hydroxymethylfurfural (HMF, 98%), 5-ethoxymethylfurfural (EMF, 97%), ethyl levulinate (EL, 99%), and zirconium octahydrate oxychloride were purchased from Macklin Biochemical Co., Ltd. Ethanol (≥99.8%), glacial acetic acid, N, N-dimethylformamide (DMF), and methylene chloride were obtained from Sinopharm Chemical Reagent Co., Ltd. Chlorosulfonic acid was purchased from Shanghai Jiuding Chemical Reagent Co., Ltd.

### Catalyst Preparation

In a 100-ml round-bottom flask, 4.8 g ZrOCl_2_·8H_2_O (15 mmol), 2.4 g terephthalic acid (15 mmol), 50 ml DMF, and 6 ml acetic acid was mixed, and the mixture was then treated with ultrasound for 30 min. After that, the resultant mixture was kept under constant stirring at 120°C for 24 h, and the obtained white precipitate was washed with methanol and DMF several times. After drying, the mashy white solids were calcined in a muffle furnace to give UIO-66 (Ragon et al., 2014).

The as-prepared UIO-66 (1 g) was dispersed in 40 ml CH_2_Cl_2_ and stirred at 0°C for 30 min. Under vigorous magnetic stirring, ClSO_3_H x mL in 10 ml CH_2_Cl_2_ was slowly added to the mixture. After the reaction continued for 4 h, the produced solid was filtered and washed with ethanol and then with deionized water and dried in a vacuum oven. The prepared catalysts are designated as UIO-66-SO_3_H-x (x = 0.3, 0.5, 0.8, and 1.0 ml), and x represents the volume of chlorosulfonic acid added.

### Fructose Degradation

The catalytic upgrading of fructose was carried out in a 50-ml autoclave with a Teflon liner. Typically, the Teflon liner was loaded with fructose (1 mmol, 0.180 g), ethanol (5 ml) as the reactant and solvent, and the UIO-66-SO_3_H catalyst. Then, the reaction mixture was heated to the prescribed temperature for a desired reaction time with stirring. After finishing the reaction, the reactor was given an ice bath for rapid cooling to room temperature.

Then, the mixture products were diluted and filtered using a 0.22-μm syringe filter. The fructose and the formed products were analyzed using an Agilent 1260 HPLC chromatograph equipped with refractive index and UV detectors. The external standard method was used to analyze the substrate conversion rate and selectivity of each product, and the calculation equation is as follows:

$$Fructose\;conversion\left(\%\right)=\left(1-\;\frac{Mole\;of\;Fructose}{Initial\;mole\;of\;Fructose}\right)\times 100\%;$$

$$EMF\;yield\left(\%\right)=\frac{Mole\;of\;EMF}{Initial\;mole\;of\;Fructose}\times 100\%;$$

$$HMF\;yield\left(\%\right)=\frac{Mole\;of\;HMF}{Initial\;mole\;of\;Fructose}\times 100\%.$$

## Results and Discussion

### Catalyst Characterization

**FIGURE 2:**
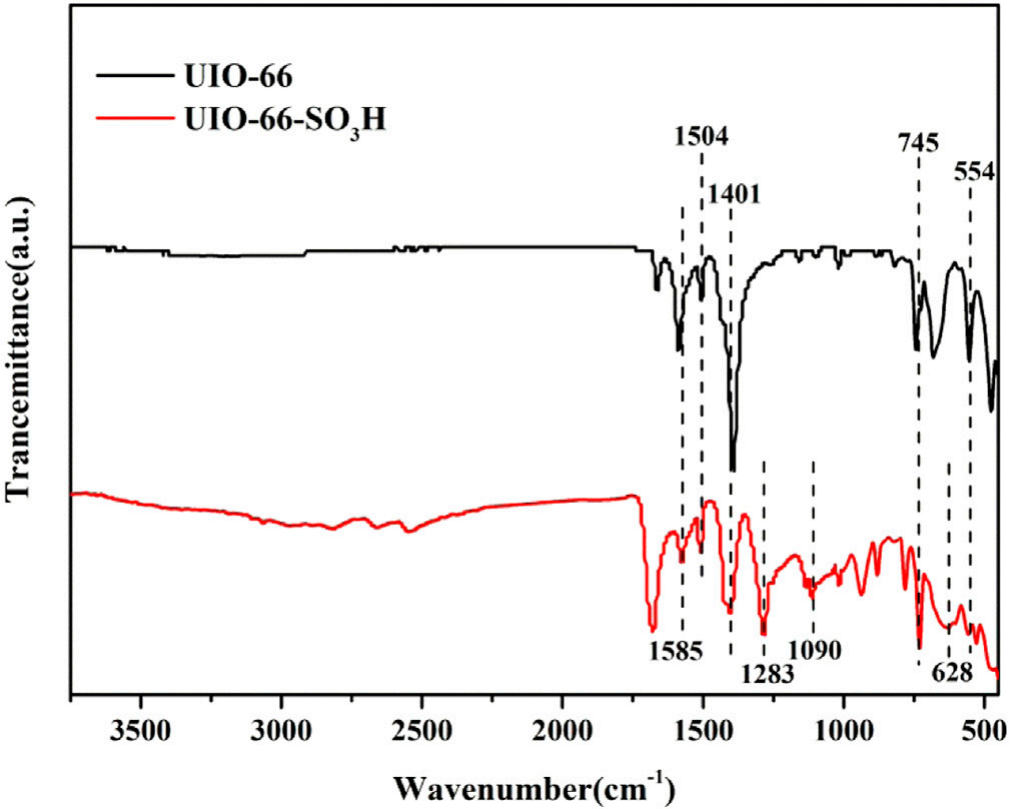
FT-IR spectra of UIO-66 and UIO-66-SO_3_H.

The FT-IR spectra of the metal–organic framework material UIO-66 and catalyst UIO-66-SO_3_H are shown in Figure 2, in which 1,585 cm^−1^ is the benzene ring skeleton stretching vibration peak; 1,504 cm^−1^ and 1,401 cm^−1^ are the absorption peaks corresponding to the structural unit (Zr_6_O_4_)(OH)_4_(CO_2_)_n_ formed by coordination of terephthalic acid with zirconium nodes (Zaboon et al., 2018). The fingerprint region 745 cm^−1^ is the out-of-plane bending vibration of C-H; an absorption peak can be seen at 554 cm^−1^, which is the Zr-O bond stretching vibration peak in the metal–organic skeleton. In the IR curve of UIO-66-SO_3_H, 1,283 cm^−1^ and 1,090 cm^−1^ belong to the O=S=O symmetric stretching vibration peak and S-O stretching vibration peak, respectively; 628 cm^−1^ is the stretching vibration peak of the C-S bond (Chen T.-F. et al., 2019). Thus, the presence of sulfonic acid groups in the UIO-66-SO_3_H catalyst was confirmed, indicating the successful preparation of sulfonic acid-functionalized materials.

**FIGURE 3:**
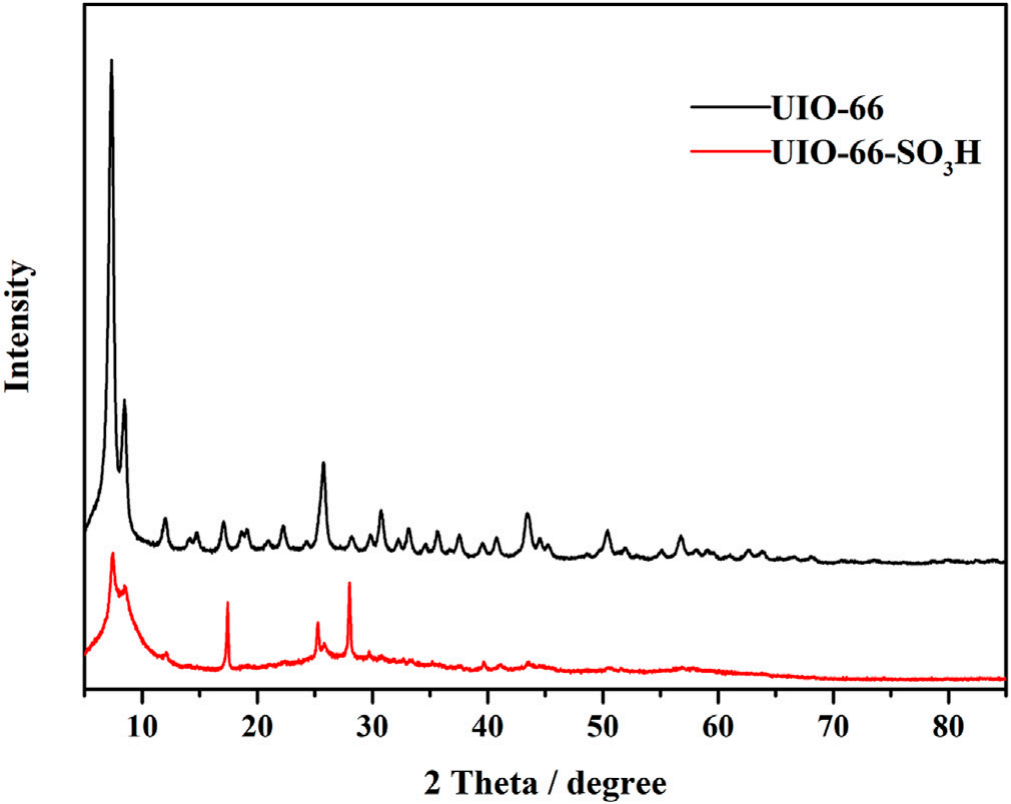
XRD patterns of UIO-66 and UIO-66-SO_3_H.

The XRD spectra of UIO-66 and UIO-66-SO_3_H are shown in Figure 3, in which the XRD spectrum of UIO-66 is consistent with that in the literature. Some XRD peaks of the UIO-66-SO_3_H catalyst were shifted, and the intensity of the peaks was significantly weakened compared with the UIO-66 sample, which is similar to the literature report. This was because during the introduction of strong Brønsted acidity into UIO-66, the chlorosulphonic acid impaired its crystallinity as well as the stability of the framework (Hu et al., 2016).

**FIGURE 4:**
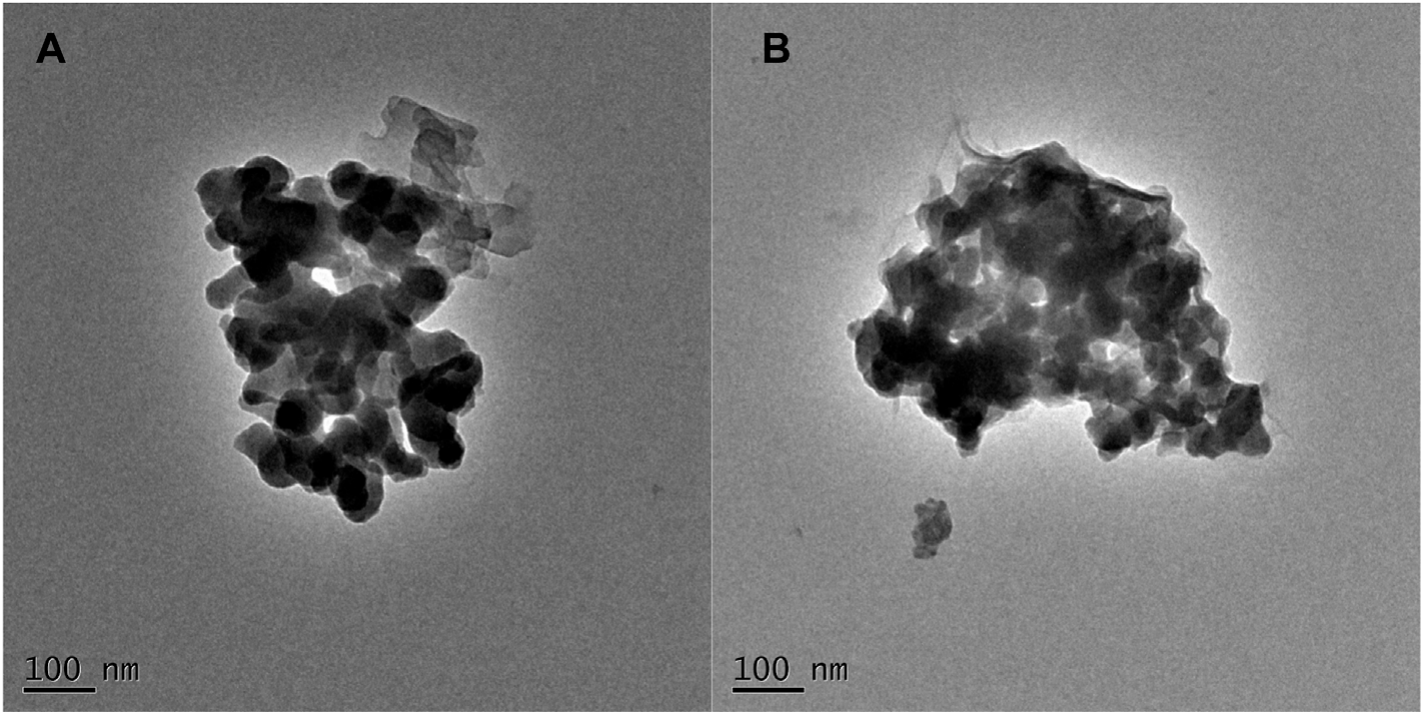
TEM image of UIO-66 **(A)** and UIO-66-SO_3_H **(B)**.

Figure 4 presents the transmission electron microscopy images of the samples, from which it can be observed that the morphology of both materials is similar and consist of particles of relatively uniform size. However, they are also in a more agglomerated state, which can be attributed to the particle size of the samples being around 50 nm, resulting in strong surface forces and agglomeration. The result of transmission electron microscopy revealed that the morphological characteristics of the UIO-66 material were hardly affected by the presence of the sulfonic acid group during the preparation of the catalyst.

### Catalytic Performance

**FIGURE 5:**
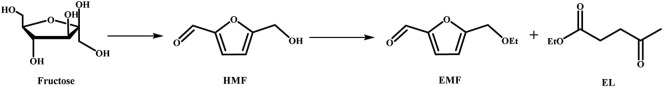
Synthesis of EMF from fructose.

In this study, ethanol was used as the reactant and reaction medium, and the reaction was carried out in the order of fructose dehydration to produce HMF and ethyl alcohol etherification to produce EMF, and the ring-opening by-product EL was also generated (Figure 5).

**FIGURE 6:**
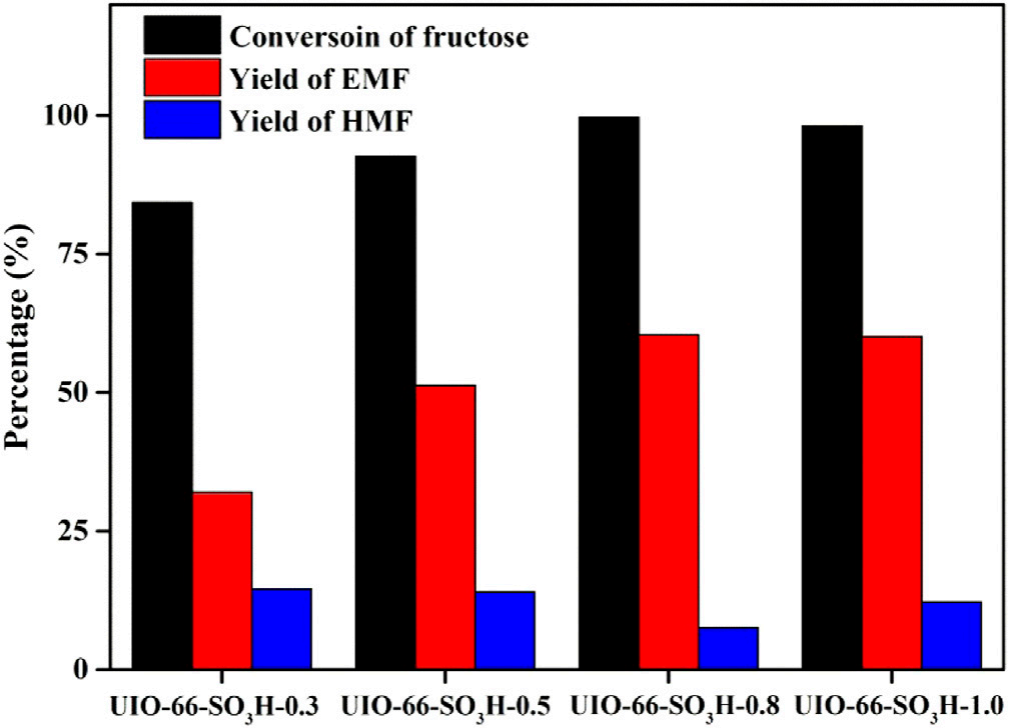
Results of alcoholysis of fructose catalyzed by UIO-66-SO_3_H with different acidity. Reaction conditions: 1 mmol fructose, 5 ml ethanol, 30 mg catalyst, 1 h, and 140°C.

For the sulfonation of the prepared metal–organic framework UIO-66, the same mass of UIO-66 added with different amounts of the sulfonating agent resulted in different acid amounts of UIO-66-SO_3_H catalysts as listed in Table 1. The UIO-66 (1 g) added with the volume of chlorosulfonic acid increased from 0.3 to 1.0 ml, and the acid amount of the catalytic material showed a roughly increasing trend. The highest acid amount of the catalyst was measured at 0.8 ml of chlorosulfonic acid, and the best catalytic effect was achieved with UIO-66-SO_3_H showing a maximum acid amount of 1.46 mmol/g. To some extent, the acid amount of the catalyst increased with increasing concentration of the sulfonating agent. However, due to the gradual increase of the spatial site resistance and the damage to the catalyst structure caused by the addition of too much chlorosulfonic acid, further increase of the sulfonating agent did not increase the amount of acid, while the sulfonated materials were all effective in catalyzing the conversion of fructose to EMF. Accordingly, the effect of the acid amount of the catalyst on the catalytic activity is reflected in Figure 6. If the catalyst material was used without sulfonic acid groups, the target product is almost absent in the reaction solution, while the sulfonated materials were all effective in catalyzing the conversion of fructose to EMF. Thus, it is evident that the acidity of the catalyst is crucial for this reaction. With the increase of the acid amount, the conversion of fructose gradually increased, and the yield of EMF also showed an increasing trend, and it can be concluded that the acid amount is closely related to the synthesis of the target product EMF yield. Based on the aforementioned conclusions, the effect of factors such as reaction temperature, time, and catalyst amount on fructose alcoholysis of UIO-66-SO_3_H was further investigated systematically in this article.

**TABLE 1:** Acid content of UIO-66-SO_3_H with different dosages of ClSO_3_H.

Entry	Sample	Acid amount (mmol/g)
1	UIO-66-SO_3_H-0.3	0.87
2	UIO-66-SO_3_H-0.5	1.13
3	UIO-66-SO_3_H-0.8	1.46
4	UIO-66-SO_3_H-1.0	1.30

**FIGURE 7:**
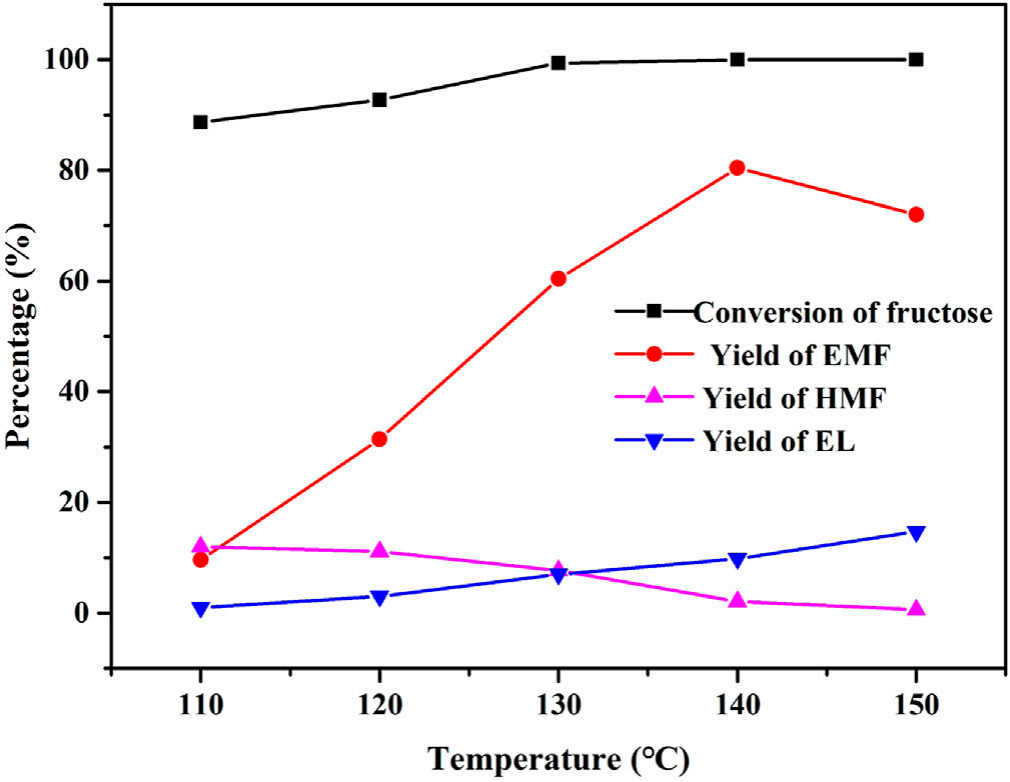
Effect of reaction temperature on the alcoholysis of fructose. Reaction conditions: 1 mmol fructose, 5 ml ethanol, 30 mg catalyst, and 1 h.

The effect of reaction temperature in the range of 110–150°C on the conversion of fructose to EMF was investigated using UIO-66-SO_3_H-0.8 as the catalyst, and the results are shown in Figure 7. As the reaction temperature was 110°C, the yield of both EMF and HMF was low, and a trace amount of EL was detected. With the increase in the reaction temperature, the fructose was gradually converted from 88.7% to complete conversion, indicating that the dehydration of fructose required higher temperature, and the etherification of HMF was easier than the dehydration of fructose into HMF. The yield of EMF increased significantly from 9.6 to 80.4% with the increase from 110 to 140°C, the yield of the intermediate product HMF decreased, and the byproduct EL showed a gradual increase. However, when the temperature was increased to 150°C, the yield of the target product EMF decreased due to the ring opening of the side reaction EMF, which might be due to the instability of EMF at higher reaction temperatures under acidic conditions, resulting in the generation of more ring-opening product EL. The increase in temperature was favorable to the conversion of fructose, and too high temperature promoted the decomposition of EMF into other products. Therefore, the optimal reaction temperature for this experiment was 140°C.

**FIGURE 8:**
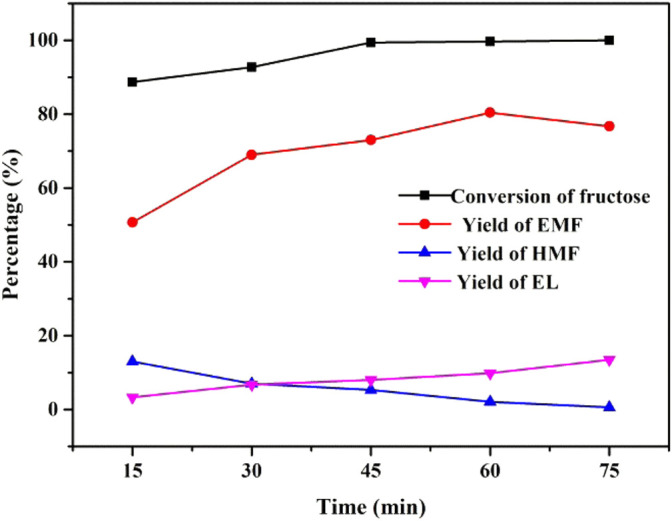
Effect of reaction time on the alcoholysis of fructose. Reaction conditions: 1 mmol fructose, 5 ml ethanol, 30 mg catalyst, and 140°C.

Figure 8 shows the effect of the reaction time of UIO-66-SO_3_H-0.8 as a catalyst on the synthesis of EMF from fructose in the ethanol system at a reaction temperature of 140°C. It can be seen that the target product EMF yield increased relatively fast at the beginning of the reaction stage, reaching 69% within 30 min, and as the reaction time continued to extend, the yield of EMF reached the best yield of 80.4% at 1 h, followed by a slight decrease in the yield of EMF. The content of HMF gradually decreased with the extension of time and was gradually converted into EMF, while the yield of EL gradually increased. This result indicates that fructose conversion is a typical continuous reaction in the ethanol reaction system. The longer reaction time allowed the conversion of EMF to its byproduct EL. Since the long time was not favorable for the production of EMF, a reaction time of 1 h was chosen for the subsequent reaction.

**FIGURE 9:**
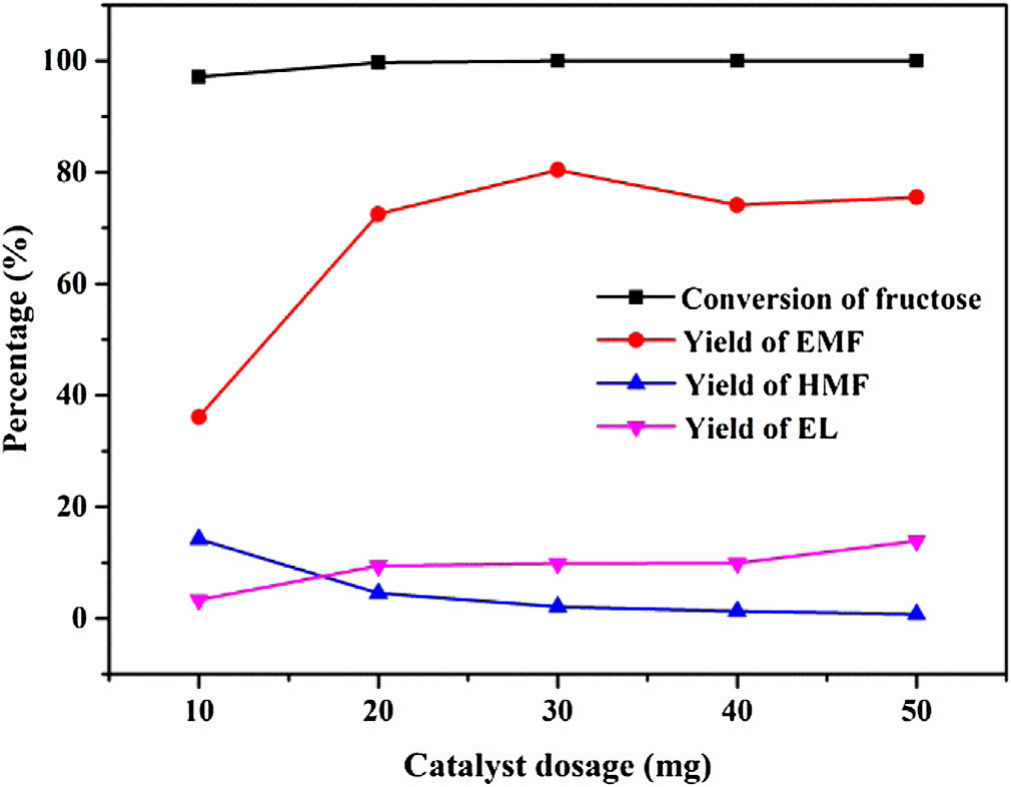
Effect of catalyst dosage on the alcoholysis of fructose. Reaction conditions: 1 mmol fructose, 5 ml ethanol, 1 h, and 140°C.

Under the conditions of 180 mg of substrate, 140°C of reaction temperature, and 1 h of reaction time, the effects of catalyst dosages of 10 mg, 20 mg, 30 mg, 40 mg, and 50 mg on the conversion of fructose to synthesize EMF were investigated. As seen in Figure 9, under the condition of 10 mg catalyst dosage, the fructose was almost completely converted, and the EMF yield was 36.1%. It can be observed in the figures that the increase of catalyst dosage from 10 to 20 mg increased the yield of EMF by about double, which was 72.5%. The highest EMF yield was obtained when the catalyst dosage was 30 mg, while the yield decreased slightly when the catalyst dosage was 40 mg and more. Therefore, 30 mg was chosen as the best catalyst dosage.

**FIGURE 10:**
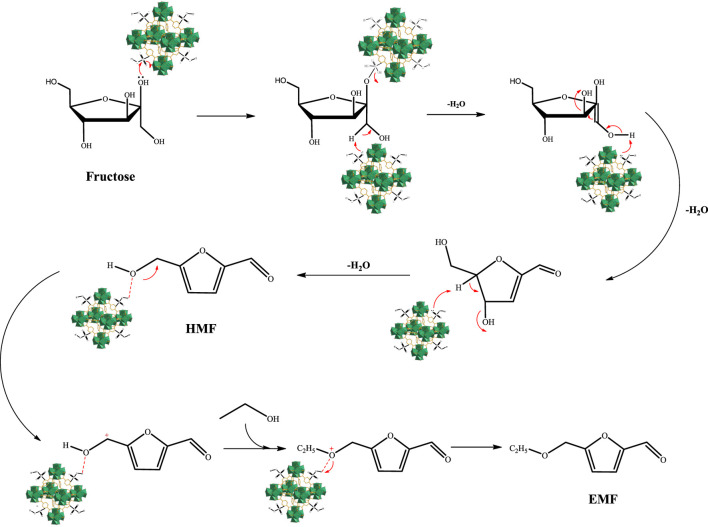
Possible reaction mechanism for the conversion of fructose to EMF in ethanol catalyzed by UIO-66-SO_3_H(Amarasekara et al., 2008; Ohara et al., 2010; Yan et al., 2022).

In addition, the possible mechanism for catalyzed conversion of fructose to EMF is shown in Figure 10. Fructose is dehydrated, dehydrogenated, and dehydrated to produce HMF under acidic conditions. Then, the acid site on the catalyst surface protonated with hydroxyl oxygen in HMF to form carbocation, followed by ethanol attack on carbocation, and finally formed EMF after proton migration (Amarasekara et al., 2008; Ohara et al., 2010; Yan et al., 2022).

### Comparison With Reported Catalysts

The literature related to the one-step conversion of fructose to EMF is presented in Table 2, which compares the catalytic activity of the catalyst prepared in this study, UIO-66-SO_3_H, with other catalysts. For example, Dai et al. (2019) reported a solid organocatalyst, double hydrogen-bonded sulfonated polymer catalyst (D-SPC), to convert fructose to EMF in 18 h at 140°C with a maximum yield of 68.8%. Manjunathan et al., (2021) found a catalyst consisting of divinylbenzene (DVB) and sodium p-styrenesulfonate (SPSS) copolymerized with a sulfonated hydrophobic mesoporous organic polymer (MOP-SO_3_H) catalyst, which was able to achieve an EMF yield of 72.2% under the conditions of a bulk catalyst-catalyzed reaction. Bharath et al., (2021) immobilized the ultra-small Pd–Ru nanoparticles on the surface of 2D MXene nanosheets to produce a catalyst (Pd-Ru/MXene), which was reacted at 120°C for 2.5 h and was able to obtain a final EMF yield of 82%. Compared with the previous two catalysts, the catalyst prepared in this study has good catalytic effect. Meanwhile, the UIO-66-SO_3_H catalyst catalyzed the conversion of fructose to EMF in this experiment under lower conditions that were easily satisfied, and the reaction time was greatly reduced to achieve high catalytic effect.

**TABLE 2:** Catalytic performance of UIO-66-SO_3_H compared to reported solid acid catalysts for the EMF synthesis from fructose.

Entry	Catalyst	Solvent	Time/h	T/°C	EMF yield/%	Reference
1	D0.5-SPC	Ethanol/THF	18	140	68.8	Dai et al. (2019)
2	MOP-SO_3_H-0.6	Ethanol	5	100	72.2	Manjunathan et al. (2021)
3	Pd-Ru/MXene	Ethanol	2.5	120	82.0	Bharath et al. (2021)
4	UIO-66-SO_3_H	Ethanol	1	140	80.4	This work

## Conclusion

In summary, we prepared the MOF material UIO-66 and functionalized it using chlorosulfonic acid under mild conditions to produce a series of sulfonic acid-functionalized MOF-based solid catalysts (UIO-66-SO_3_H). The catalyst was characterized, and the corresponding results showed that the sulphonic acid group was successfully bonded to the benzene ring on the metal–organic framework. In addition, the acid amount of the catalyst could be adjusted by changing the amount of chlorosulfonic acid, and the highest acidity of the catalyst reached 1.46 mmol/g. The as-prepared heterogeneous catalyst UIO-66-SO_3_H showed efficient catalytic performance for the one-pot conversion of fructose to biofuel EMF under cosolvent-free conditions. The effect of different reaction conditions on the catalytic reaction was systematically investigated, and the EMF yield of 80.4% could be obtained under the optimum reaction conditions. Compared with those previously reported, the catalyst prepared in this work has the advantages of easy preparation, mild reaction conditions, and a good catalytic effect. It is expected that our catalytic system can be further applied for the efficient production of biofuel EMF in industrial applications.

## Data Availability

The original contributions presented in the study are included in the article/Supplementary Material, further inquiries can be directed to the corresponding authors.
